# Acute kidney injury in an elderly patient with presumed acute focal bacterial nephritis: a case report highlighting diagnostic challenges

**DOI:** 10.3389/fmed.2026.1948946

**Published:** 2026-10-22

**Authors:** Qi Li, Daorina Bao, Haidong Zhang, Zhuan Cui, Yao Yao, Min Lu, Danxia Zheng, Yue Wang

**Affiliations:** 1 Department of Nephrology, Peking University Third Hospital, Beijing, China; 2 Department of Pathology, Peking University Third Hospital, Beijing, China

**Keywords:** acute focal bacterial nephritis, acute kidney injury, hemodialysis, IgA-dominant infection-related glomerulonephritis, renal biopsy

## Abstract

Acute focal bacterial nephritis (AFBN) is often characterized as a complicated form of upper urinary tract infection. Its relationship with acute kidney injury (AKI) is not well established, and most reported cases have preserved renal function because most patients respond well to antibiotic treatment. We report a 74-year-old woman with type 2 diabetes mellitus who presented with fever, diarrhea, oliguria, and severe AKI requiring hemodialysis. Blood and urine next-generation sequencing identified *Escherichia coli*. Imaging showed bilateral renal enlargement and cortical swelling suggestive of presumed AFBN. Despite 4 weeks of antibiotic therapy, renal function failed to recover. Empirical corticosteroid therapy transiently increased urine output but did not improve serum creatinine. Renal biopsy revealed IgA-dominant infection-related glomerulonephritis (IgA-IRGN) with tubulointerstitial injury. She remained dialysis-dependent 1 year later. This case illustrates the complexity of AKI in a patient with sepsis, presumed AFBN, and IgA-IRGN. We highlight the importance of considering multiple etiologies and the role of renal biopsy when renal function fails to recover after infection control.

## Introduction

Acute focal bacterial nephritis (AFBN), also known as acute lobar nephronia, is considered a complicated upper urinary tract infection that lies between acute pyelonephritis and acute renal abscess (1, 2). The disease has mainly been reported in pediatric patients. Few reports have described that adult AFBN typically presents with hematuria, flank pain, and symptoms of bladder irritation, and the renal function generally remains within the normal range (3–6). Renal insufficiency in AFBN patients often recovers favorably following longer durations of antibiotic treatment (7, 8). To date, reports of AFBN complicated by acute kidney injury (AKI) have been rare. Herein, we describe an elderly woman with presumed AFBN and severe AKI, in whom kidney biopsy findings favored a diagnosis of IgA-IRGN. This report aims to highlight the diagnostic challenges when AKI complicates presumed AFBN, particularly in elderly diabetic patients. It underscores the need to investigate alternative or coexisting causes of renal impairment and the critical role of renal biopsy in guiding management when renal function fails to recover after appropriate antimicrobial therapy.

## Case report

### Medical history

A 74-year-old woman developed a fever 2 weeks before admission following a long-haul flight (peak temperature 38.8°C), accompanied by nausea, diarrhea, and loss of appetite. Her fever and diarrhea symptoms were alleviated after taking acetaminophen and rifaximin. One week before admission, she developed oliguria (350 mL/day) and dyspnea. The symptoms progressed, leading to her hospitalization 1 day ago due to deteriorating dyspnea.

On admission, physical examination showed sinus tachycardia (106 beats/min), normothermia (36.5°C), normotension (130/56 mmHg), and tachypnea (respiratory rate 28 breaths/minute, arterial oxygen saturation of 97% on room air), with bilateral pulmonary crackles, moderate pitting edema in the lower limbs, and dullness on bladder percussion. There was no abdominal tenderness, rebound tenderness, or renal tenderness.

Her past medical history included hypertension for 10 years, hyperlipidemia for 10 years, and type 2 diabetes mellitus (T2DM) for 20 years. She was being treated with felodipine, atorvastatin, and aspirin, along with metformin and insulin injections for glucose control.

### Laboratory examination and imaging

Laboratory examination revealed pyuria and elevated inflammatory markers and serum creatinine levels. Based on the findings of hematuria and proteinuria, a differential diagnosis was conducted to exclude secondary causes, including lupus, vasculitis, and hematological malignancies (Table 1). The pathogen was confirmed as *Escherichia coli* through next-generation sequencing (NGS) of blood and urine samples collected the day after admission. Contrast-enhanced computed tomography (CT) was not performed due to the patient's AKI. MRI was also not performed during the acute phase, as her clinical instability precluded safe transport to the MRI suite and completion of the examination. Non-contrast abdominal CT revealed bilateral renal enlargement with marked cortical thickening, more pronounced on the left, along with an ill-defined area of relative hypoattenuation in the upper-pole parenchyma of the left kidney (Figure 1). Ultrasonography also revealed bilateral renal enlargement with substantial cortical swelling (left kidney size 13.6 × 6.5 × 5.8 cm, maximum cortical swelling thickness of 3.0 cm; right kidney size 13.3 × 6.2 × 5.5 cm), without evidence of hydronephrosis or abscess formation.

**Table 1:** Laboratory examination results of the patient.

Blood test	Results	Reference range	Immune and inflammatory tests	Results	Reference range	Urine test	**Results**	Reference range
WBC	**52** **.** **85↑**	4–10 × 10^9^/L	Total complement	**62.8↑**	23–62 U/mL	FCM-WBC	**41,905↑**	0–30/μL
Neut%	**97**.**8↑**	40%–75%	IgA	**6.15↑**	1.0–4.2 g/L	FCM-RBC	**15,125↑**	0–25/μL
HGB	**112↓**	115–150 g/L	IgG	**19.1↑**	8.6–17.4 g/L	FCM-BACT	**7,698↑**	≤6,000/μL
PLT	192	125–350 × 10^9^/L	PCT	**5.9↑**	<0.1 ng/mL	M-WBC	**Full field of vision**	NA
SCr	**689↑**	53–97 μmol/L	CRP	**96↑**	0–10 mg/L	M-RBC	**Full field of vision**	NA
SCr baseline	65	53–97 μmol/L	C3	0.913	0.7–1.4 g/L	UPCR	**1,655↑**	<200 mg/g.Cr
BUN	**66**.**3↑**	3.1–8.8 mmol/L	C4	0.316	0.1–0.4 g/L	UACR	**415.3↑**	≤30 mg/g.Cr
Potassium	**7**.**11↑**	3.5–5.3 mmol/L	ANA	**1:80**	<1:40	24h-UTP	**598↑**	<150 mg
Phosphate	**3**.**98↑**	0.85–1.51 mmol/L	Anti-dsDNA antibody	0.44	<20 IU/mL	pH	**6.0**	5.5–6.5
HbA1c	**8**.**7↑**	4.0%–6.0%	Anti-GBM antibody	Negative	<1:10	SG	**1.006↓**	1.015–1.025
Calcium	**1**.**86↓**	2.2–2.7 mmol/L	ANCA	Negative	NA	Urine NGS	***Escherichia coli***	NA
ALB	**28**.**5↓**	35–50 g/L	Serum IFE	Normal	NA			
CO_2_CP	**5**.**5↓**	21–31 mmol/L	Urinary IFE	Normal	NA			
Blood NGS	***Escherichia coli***	NA						

ALB, albumin; ANA, anti-nuclear antibody; ANCA, anti-neutrophil cytoplasmic antibody; BUN, blood urea nitrogen; BACT, bacteria; CO_2_CP, carbon dioxide combining power; CRP, C-reactive protein; dsDNA, double-stranded DNA; FCM, flow cytometry; GBM, glomerular basement membrane; HGB, hemogloblin; HbA1c, hemoglobin A1c; IFE, immunofixation electrophoresis; M, microscopic; Neut, neutrophil; NGS, next-generation sequencing; NA, not applicable; PLT, platelet; PCT, procalcitonin; RBC, red blood cell; SCr, serum creatinine; SG, specific gravity; UACR, urinary albumin-to-creatinine ratio; UPCR, urinary protein-to-creatinine ratio; UTP, urinary total protein; WBC, white blood cell. NGS results were classified as high confidence by the validated internal analytical pipeline of the hospital laboratory. *Escherichia coli* was detected in whole blood (5,033 specific reads) and urine (2,522 specific reads). Values outside the reference range are shown in bold. Upward and downward arrows indicate values above and below the reference range.

**Figure 1:**
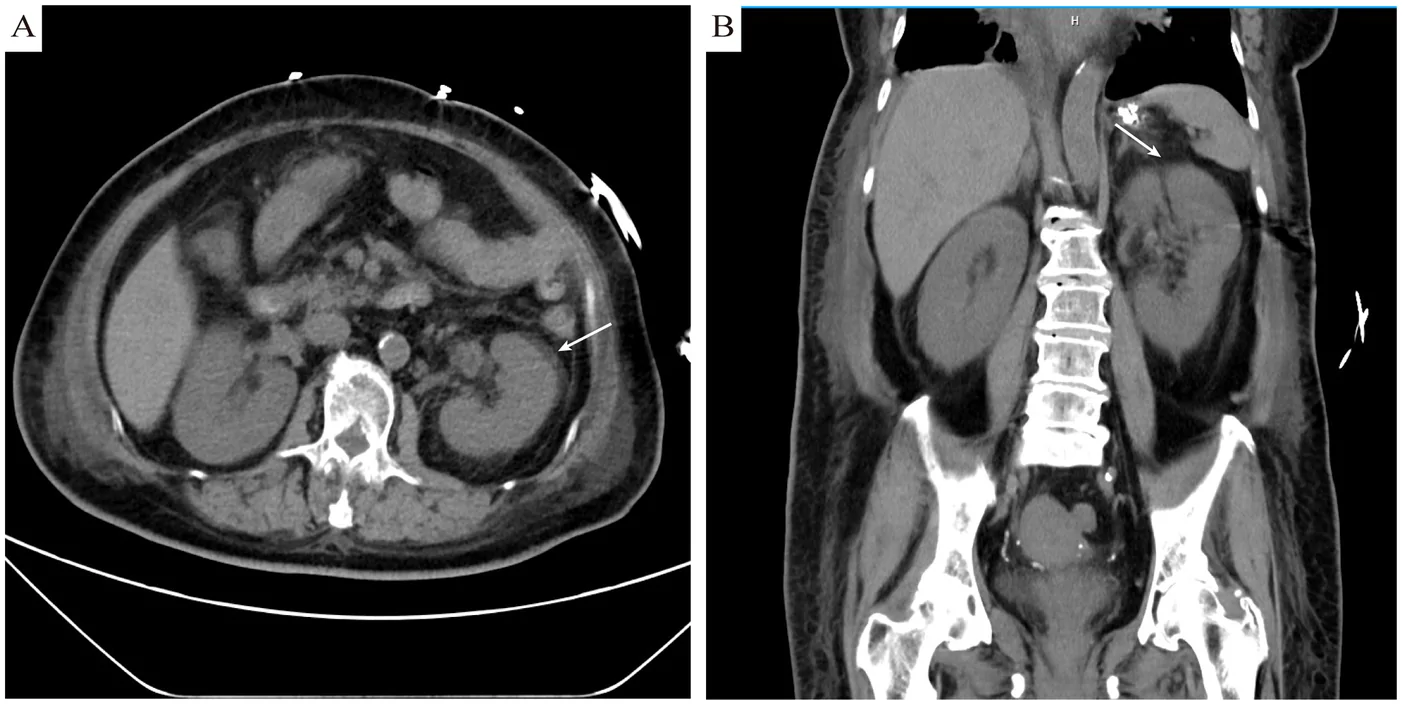
Computed tomography scan of the abdominal-pelvic compartment of this patient on admission. **(A)** Axial and **(B)** coronal images show bilateral renal enlargement and cortical thickening, with an ill-defined area of relative hypoattenuation in the upper-pole parenchyma of the left kidney (arrows).

### Diagnosis, treatment, and prognosis

The identification of *Escherichia coli* by NGS supported the presence of both an upper urinary tract infection and a bloodstream infection. Given an acute increase of at least four points in the SOFA score, the patient met the criteria for sepsis. In conjunction with the clinical presentation and bilateral renal enlargement with cortical swelling demonstrated by CT and ultrasonography, the overall evidence supported a diagnosis of presumed AFBN.

Upon admission, hemodialysis was performed due to AKI with oliguria and hyperkalemia. Antibacterial treatment using intravenous administration of meropenem (1 g every 12 h) was initiated based on NGS identification and clinical presentation. The dosage was adjusted according to the creatinine clearance, and on hemodialysis days, the scheduled dose was administered after the completion of the dialysis session. After 4 weeks of antibiotic therapy, blood NGS became negative, FCM-WBC dropped to 573/μL, FCM-BACT dropped to 219/μL, and PCT dropped to 0.105 ng/mL. Ultrasonography showed resolution of left renal cortical swelling (left kidney size 13.5 × 7.4 × 5.0 cm, cortical thickness 1.2 cm; right kidney 12.6 × 6.2 × 5.7 cm, cortical thickness 0.9 cm). Despite infection control, urine output increased to approximately 1,000 mL/day with low-dose diuretics; however, renal function did not recover. Given persistent leukocyturia and suspected acute interstitial nephritis, renal biopsy was performed on the same day that empirical corticosteroid treatment was initiated. Methylprednisolone (24 mg/day, p.o.) was given for a 14-day course. During this period, urine output increased to over 2,000 mL/day without diuretics, but there was no corresponding biochemical recovery of renal function.

The renal biopsy showed granular deposition of IgG (1+), IgA (3+), IgM (segmentally 2+), C3 (3+), *κ* (3+), and *λ* (3+) along mesangial and capillary walls. Light microscopic examination revealed that 2/9 glomeruli were ischemic and sclerotic. The remaining glomeruli exhibited mild, diffuse mesangial expansion, with focal and segmental accentuation accompanied by endothelial cell proliferation. Fuchsinophilic deposits were observed in the subepithelial and mesangial regions, with occasional neutrophil infiltration. Tubules displayed acute injury with epithelial simplification and small foci of tubular atrophy. Protein casts and crystalloid structures were identified within tubular lumina. The interstitium exhibited edema, accompanied by small foci of lymphocytic, mononuclear, and neutrophilic infiltration, along with early fibrosis. Small arteries demonstrated wall thickening. Electron microscopy showed mesangial hypercellularity and matrix expansion accompanied by subendothelial and mesangial electron-dense deposits. Extensive effacement of podocyte foot processes was also noted (Figure 2). The combination of endocapillary proliferation, neutrophil infiltration, and granular IgA/C3 deposition in the setting of recent *Escherichia coli* infection was most consistent with IgA-dominant infection-related glomerulonephritis (IgA-IRGN) accompanied by tubulointerstitial injury.

**Figure 2:**
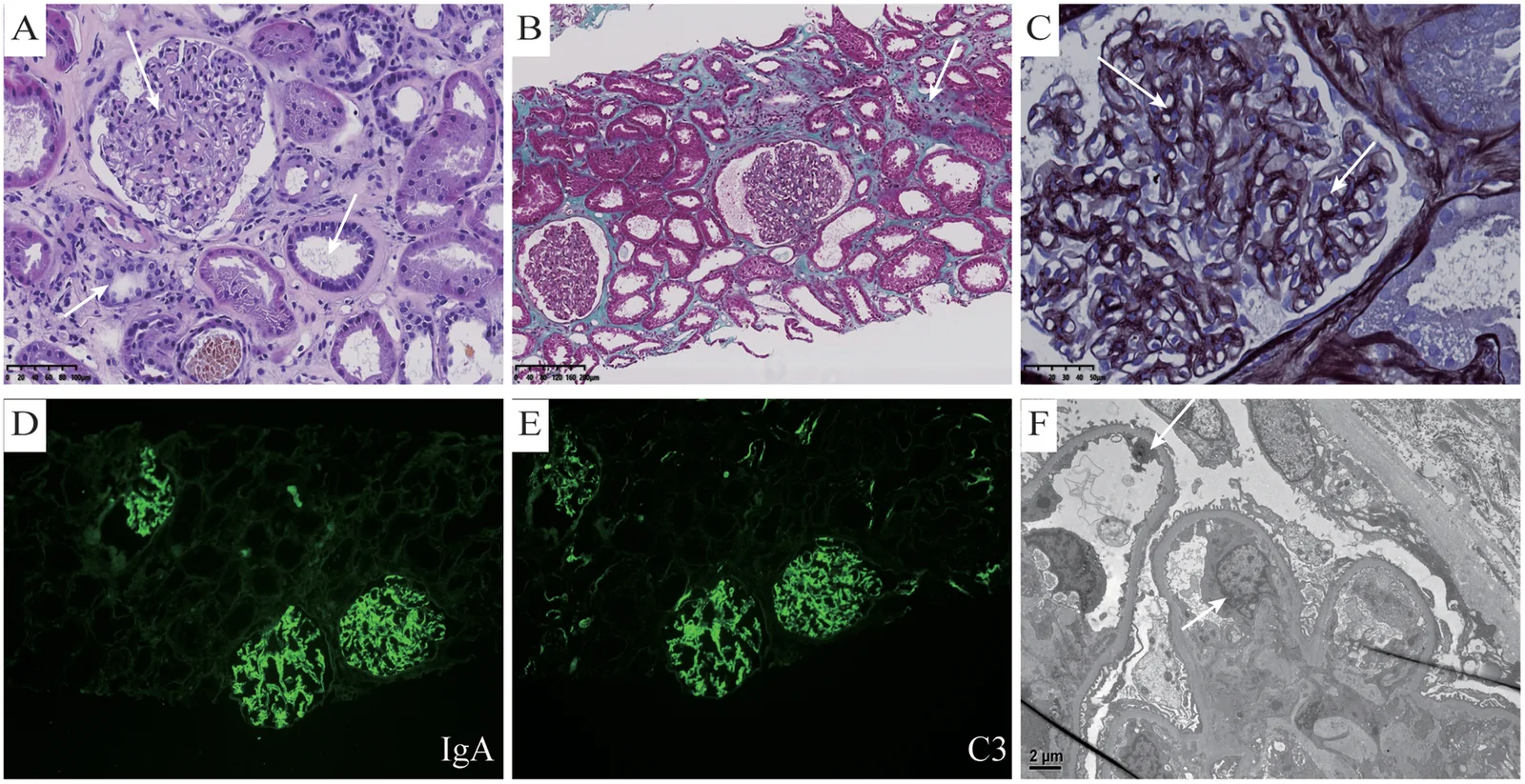
**(A)** Hematoxylin–eosin staining (original magnification ×200) and **(B)** Masson staining showed mesangial hypercellularity, dilatation of the tubular lumen, interstitial edema, and fibrosis in the tubulointerstitium (original magnification ×100). **(C)** Periodic acid-silver methenamine staining (original magnification ×400) of the renal biopsies demonstrated mesangial and endocapillary proliferation. **(D,E)** Direct immunofluorescence indicated the granular deposition of IgA and C3 (original magnification ×200). **(F)** Electron microscopy image of renal biopsy sample showed electron-dense deposits in the mesangial and subendothelial areas and fusion of the foot process in the glomeruli (original magnification ×5,000).

Meropenem was discontinued after the 4-week course. Corticosteroids were subsequently tapered off once the biopsy result favored IgA-IRGN. At discharge, the patient's urine volume had recovered to 1,500 mL/day without diuretics (Figure 3). However, she remained hemodialysis-dependent, with serum creatinine persistently elevated at approximately 500 μmol/L during the 1-year follow-up period.

**Figure 3:**
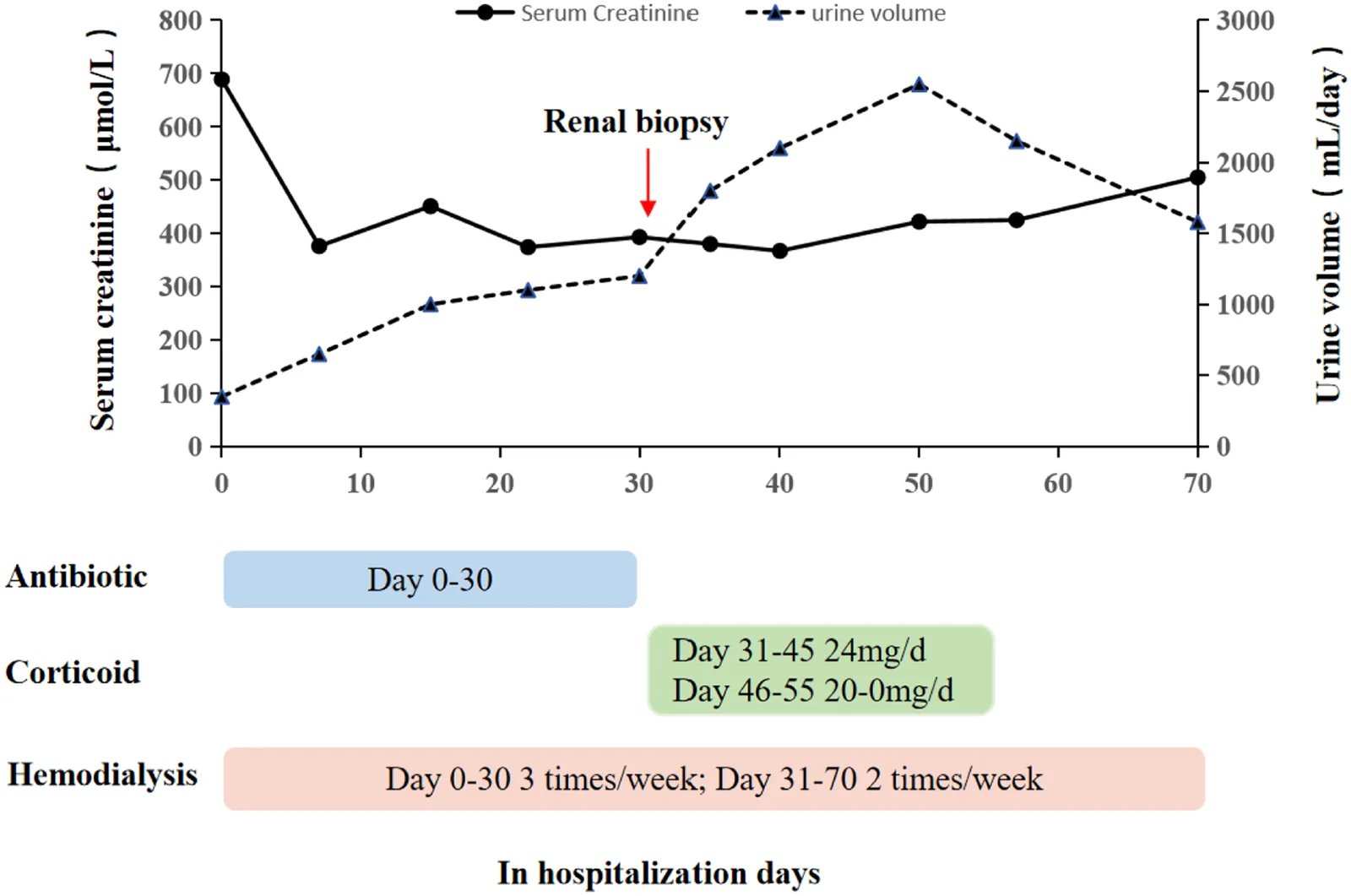
Clinical course of the patient during hospitalization.

## Discussion

AFBN typically presents with high fever, flank or abdominal pain, symptoms of urinary tract irritation, and pyuria, with or without renal insufficiency (1, 9), mimicking the clinical onset of acute pyelonephritis. Hematogenous dissemination of bacteria, including *Escherichia coli*, *Enterococci*, *Staphylococcus aureus*, and *Klebsiella*, through segmental renal arteries is a major pathogenic mechanism in the development of AFBN (10–13). The diagnosis of AFBN is established by the presence of wedge-shaped renal hypodense lesions in contrast-enhanced CT or MRI that typically regress after a course of anti-infection therapy (4). However, in patients with AKI in whom contrast administration is contraindicated, non-contrast CT and ultrasonography can also provide valuable diagnostic clues. Diffusion-weighted MRI, including diffusion-weighted whole-body imaging with background body signal suppression (DWIBS), has been reported as a useful alternative for diagnosing AFBN when contrast-enhanced CT is not feasible (14). Common predisposing factors for AFBN include T2DM, urolithiasis, urinary tract anomalies, pregnancy, preceding respiratory infection, kidney transplantation, substance abuse, and autoimmune diseases (5, 8, 15–17).

In the present case, the patient was at high risk for AFBN given her long-standing T2DM and advanced age. The pathogenesis of AKI in this patient was likely multifactorial. The initial diarrheal episode probably contributed to hypovolemia, which, together with subsequent *E. coli* sepsis and AFBN, may have each played a role in the development of renal injury. The relative contribution of each factor to AKI could not be clearly distinguished, as they were closely intertwined in the clinical course. Clinically, distinguishing between simple acute tubular necrosis and drug- or infection-related acute interstitial nephritis is challenging. In patients with acute pyelonephritis or AFBN complicated by AKI, early intervention with antibiotic therapy and potentially adjunctive corticosteroids, may be associated with favorable renal outcomes. Notably, approximately 40% of patients with pyelonephritis-related AKI may recover normal renal function with appropriate treatment (18). AFBN patients usually require a prolonged course of anti-infection therapy (empirically 3–4 weeks in adult patients) until renal hypodense lesions resolve on enhanced CT (5, 7, 8, 19–21), and to avert serious complications, such as renal abscess or fibrosis (20, 21). In the present case, despite 4 weeks of antibiotic therapy resulting in infection control (clearance of *E. coli* by NGS, normalization of inflammatory markers, and resolution of cortical swelling on imaging), renal function failed to recover. To our knowledge, only two cases of AKI secondary to AFBN have been reported, both exhibiting a favorable prognosis. Interstitial nephritis may play a more significant role in its pathogenesis (7, 8). Empirical corticosteroid therapy was therefore initiated based on a suspicion of acute interstitial nephritis. However, the suboptimal response to corticosteroids, with only a transient increase in urine output but no improvement in serum creatinine, prompted consideration of additional pathology beyond simple acute tubulointerstitial nephropathy.

Renal biopsy demonstrated focal segmental endocapillary proliferation with occasional neutrophilic infiltration, strong granular IgA and C3 deposition (both 3+) in the mesangium and along the glomerular capillary walls, and mesangial and subendothelial electron-dense deposits. In the setting of a temporally associated, microbiologically documented *Escherichia coli* infection, this constellation of clinicopathological findings favored a diagnosis of presumed IgA-IRGN (22, 23). Importantly, the patient had documented normal renal function 6 months prior to admission, indicating that the current episode was an acute event rather than a progression of pre-existing chronic kidney disease. Previous reports have identified E. coli among the pathogens associated with biopsy-confirmed IgA-IRGN (24, 25). IgA-IRGN commonly presents with critical AKI requiring hemodialysis and is associated with high rates of progression to chronic kidney disease and death (26). Administration of corticosteroids and immunosuppressants showed no significant benefit in improving renal outcomes and reducing mortality in IgA-IRGN. Moreover, such treatment carries the risk of exacerbating infection and triggering recurrence (26, 27). Risk factors associated with poor prognosis in IgA-IRGN include elevated serum creatinine and uric acid levels, as well as pathological lesions such as crescents, endocapillary proliferation, tubulointerstitial fibrosis, and tubular atrophy (23). The presence of these poor prognostic factors in our patient—particularly the severe AKI requiring dialysis and endocapillary proliferation—likely contributed to her progression to dialysis-dependent chronic kidney disease.

Several limitations of this case should be acknowledged. First, contrast-enhanced CT was not performed due to AKI, and MRI could not be completed because the patient's critical condition during the acute phase precluded safe transport and examination. Typical wedge-shaped hypodense lesions were not identified on non-contrast CT; therefore, a diagnosis of presumed AFBN was made. Second, conventional blood and urine cultures were negative. Both samples were collected after a single 0.5 g intravenous dose of meropenem administered on the day of admission, which may have reduced the culture yield. Antimicrobial susceptibility testing was therefore unavailable, and treatment was empirical based on NGS results. Third, although the patient reported no abnormalities on previous routine urinalyses and had a normal serum creatinine level at our hospital 6 months before admission, pre-admission urinalysis results and serum IgA levels were unavailable. The clinical history and kidney biopsy findings favored IgA-IRGN; however, pre-existing IgA nephropathy could not be definitively excluded.

In conclusion, we present a rare case of presumed AFBN-associated AKI in which persistent renal dysfunction after infection control led to the diagnosis of IgA-IRGN. This case highlights the complexity of multifactorial AKI and underscores the importance of recognizing the atypical phenotype of AFBN. When AKI persists despite adequate infection control, clinicians should consider superimposed glomerulonephritis and determine the timing of renal biopsy judiciously to avoid misdiagnosis and unnecessary interventions and to guide individualized management.

## Data Availability

The original contributions presented in the study are included in the article/Supplementary Material, further inquiries can be directed to the corresponding author.
